# A Comprehensive Narrative Review of Drug Interactions Between Traveler’s Diarrhea Medications and Chronic Therapies: Implications for Clinical Practice

**DOI:** 10.7759/cureus.70213

**Published:** 2024-09-25

**Authors:** Swetha B, Rajappan Chandra Satish Kumar, Mothishwaran B, Kripa Shankar C K

**Affiliations:** 1 Department of Pharmacy Practice, SRM Institute of Science and Technology, Chennai, IND; 2 Clinical Trial and Research Unit, Interdisciplinary Institute of Indian System of Medicine, SRM Institute of Science and Technology, Chennai, IND; 3 Sri Jayendra Saraswathi Ayurveda College and Hospital, Sri Chandrasekharendra Saraswathi Viswa Mahavidyalaya, Kancheepuram, IND

**Keywords:** antidiabetic, antihypertensive, cardioprotective, drug interaction, traveler’s diarrhea

## Abstract

Diarrhea is a common illness for travelers. Traveler’s diarrhea is typically defined as experiencing at least three unformed stools per day during a stay abroad or within 10 days of returning from the destination. In this review, we consulted five databases, namely, Medicine Complete, Medscape, Drugs.com, Epocrates, and DDInter, to conduct a comprehensive drug interaction analysis. We selected commonly prescribed medications used for the treatment of traveler’s diarrhea, including ciprofloxacin, levofloxacin, norfloxacin, ofloxacin, azithromycin, rifaximin, bismuth salicylate, and loperamide. The antidiabetic medications chosen included metformin, glipizide, glimepiride, sitagliptin, linagliptin, dapagliflozin, empagliflozin, and acarbose. The chosen antihypertensive drugs were telmisartan, olmesartan, amlodipine, nifedipine, enalapril, ramipril, metoprolol, and propranolol. Aspirin, clopidogrel, ticagrelor, rivaroxaban, warfarin, atorvastatin, and rosuvastatin were also chosen as they play an essential role in cardiovascular treatment. We performed comprehensive interaction checks across all five databases for each combination of a traveler’s diarrhea medication and medication from one of the three comorbid conditions (antidiabetic, antihypertensive, or cardioprotective). We categorized the severity of interactions as mild, moderate, or severe. Similarly, we used colors to highlight the number of databases reporting drug interactions, providing insights into the reliability of these interactions across sources. Interactions with antidiabetic drugs revealed that fluoroquinolones and sulfonylureas produce severe interaction effects. Comparatively, rifaximin can be safer as it exhibited mild interaction only with metformin, whereas the other antidiabetic drugs showed no interaction effect. Levofloxacin was found to be the safest drug among hypertensive individuals as it exerted no interaction effects with any of the antihypertensive medications. Levofloxacin and rifaximin were considered to be safe as these drugs interacted with only two cardioprotective drugs. This review features the importance of a precise approach in prescribing medications for traveler’s diarrhea, especially for patients with chronic comorbidities. These findings play a pivotal role in improving awareness and providing tailored treatment for the interaction to ensure patient well-being.

## Introduction and background

Diarrhea is a common illness for travelers. Traveler’s diarrhea is typically defined as experiencing at least three unformed stools per day during a stay abroad or within 10 days of returning from the destination. Additionally, at least one of the following symptoms should be present: nausea, vomiting, abdominal cramps, a rise in temperature, melena, or fecal urgency. Although traveler’s diarrhea is not a serious condition, it can lead to hospitalization in some cases, as well as interruptions of regular activities. Traveler destinations are the major risk factor for this illness [1]. The causative agent for traveler’s diarrhea is bacteria, as it accounts for about 90% of the cases. Various types of bacteria are responsible for this illness. Among these, *Escherichia coli* is the most common etiological factor for traveler’s diarrhea [2]. The frequency of traveler’s diarrhea episodes varies among travelers, ranging from 10% to 70%. The risk of having one episode of traveler’s diarrhea rises as the duration of the journey expands; however, the incidence is highest during the first week and decreases subsequently. The incidence differs according to the destination. The diagnosis of traveler’s diarrhea relies on self-observed signs [3]. The risk of traveler’s diarrhea is greater in Africa, Asia, and South America. Travelers with immunosuppression and decreased gastric acidity are more prone to experience traveler’s diarrhea [4]. This review article aims to investigate the interactions between drugs used for traveler’s diarrhea and those used for comorbid conditions.

Therapeutic management of traveler’s diarrhea

Standard therapeutic regimens are available for treating traveler’s diarrhea based on the severity. Therapeutic regimens can be considered for both prevention and management. Individual dietary changes and chemoprophylaxis serve as the prevention for traveler’s diarrhea, whereas antisecretary agents along with antibiotics serve as the management [5]. Antibiotics such as fluoroquinolones, rifaximin, and azithromycin help manage this condition. The most used fluoroquinolones are ciprofloxacin, levofloxacin, norfloxacin, and ofloxacin. Microbial resistance has been developed for ciprofloxacin, and levofloxacin leads to a progression of prulifloxacin (a prodrug of ulifloxacin). Azithromycin is the preferred antibiotic for pediatric use. Antibiotics are not preferable in terms of prophylaxis in mild cases; rather, they can be used in high-risk cases. Bismuth salicylate and loperamide are the best choices for treating mild-to-severe cases of traveler’s diarrhea. When the condition is severe, rifaximin is preferred. Probiotics such as *Lactobacillus*, *Bifidobacterium*, and *Saccharomyces* species have also been used as additional therapy in the management of this condition [6,7]. Racecadotril, crofelemer, and zaldaride have shown beneficial results in treating traveler’s diarrhea. Although these medications may be accessible in certain foreign countries, they are not recommended due to insufficient evidence [8]. Chronic illnesses such as type 2 diabetes mellitus, hypertension, and cardiovascular diseases are common among individuals. Most of the population is prescribed antidiabetic, antihypertensive, and cardioprotective medications as lifesaving drugs. Before treating traveler’s diarrhea, it is crucial to examine the potential interactions between the prescribed medications and those the patient is currently taking. This is mandatory as drug interactions can inhibit or induce the activity of the other drugs. This review comprehensively analyzes the interactions between recommended drugs for traveler’s diarrhea and antidiabetic, antihypertensive, and cardioprotective medications. By exploring the interactive effects and management of these drug classes, we aim to provide healthcare professionals with adequate knowledge to optimize the therapeutic regimen so that both the treatment for traveler’s diarrhea and the comorbid medications produce the desired effects.

## Review

Methodology

We consulted five databases, namely, Medicine Complete, Medscape, Drugs.com, Epocrates, and DDInter, to conduct a comprehensive drug interaction analysis. We selected commonly prescribed medications used for the treatment of traveler’s diarrhea, including ciprofloxacin, levofloxacin, norfloxacin, ofloxacin, azithromycin, rifaximin, bismuth salicylate, and loperamide. We selected these medications due to their effectiveness and regular use. We selected three categories of medications in terms of comorbid conditions, including antidiabetic, antihypertensive, and cardioprotective drugs. Antidiabetic medications included metformin, glipizide, glimepiride, sitagliptin, linagliptin, dapagliflozin, empagliflozin, and acarbose. These encompass a broad range of antidiabetic drug classes. The chosen antihypertensive drugs were telmisartan, olmesartan, amlodipine, nifedipine, enalapril, ramipril, metoprolol, and propranolol. Aspirin, clopidogrel, ticagrelor, rivaroxaban, warfarin, atorvastatin, and rosuvastatin were also chosen as they play an essential role in cardiovascular treatment. We analyzed comprehensive interaction checks across all five databases for each combination of a traveler’s diarrhea medication and medication from one of the three comorbid conditions (antidiabetic, antihypertensive, or cardioprotective). We selected one drug for traveler’s diarrhea and another for the comorbid condition. We checked drug interactions between the two drugs in Medicine Complete, followed by Medscape, Drugs.com, Epocrates, and DDInter. We similarly repeated this process for all combinations of traveler’s diarrhea drugs and antidiabetic, antihypertensive, and cardioprotective drugs. The databases were searched individually for each pair of drugs to find potential drug-drug interactions. Databases displayed the severity, mechanism, and management of each interaction. The severity and frequency of the interactions can be used to identify potential interactions. We cross-verified the interaction data by examining the identical drug pair in each of the other four databases after finding possible interactions in one database. In addition to ensuring consistency, this cross-verification procedure offered information on the reliability of the interaction data. We categorized the severity of interactions as mild, moderate, or severe, and represented them with various symbols in the tables. Similarly, we used different colors to highlight the number of databases reporting drug interactions, providing insights into the reliability of these interactions across sources. If the severity of interactions between two drugs was found to be severe and appeared in more than three databases, it was considered a potential drug interaction. The interpretation of the data entailed the severity and frequency of the interactions, as well as their possible impact on patient care.

Results

**Figure 1:**
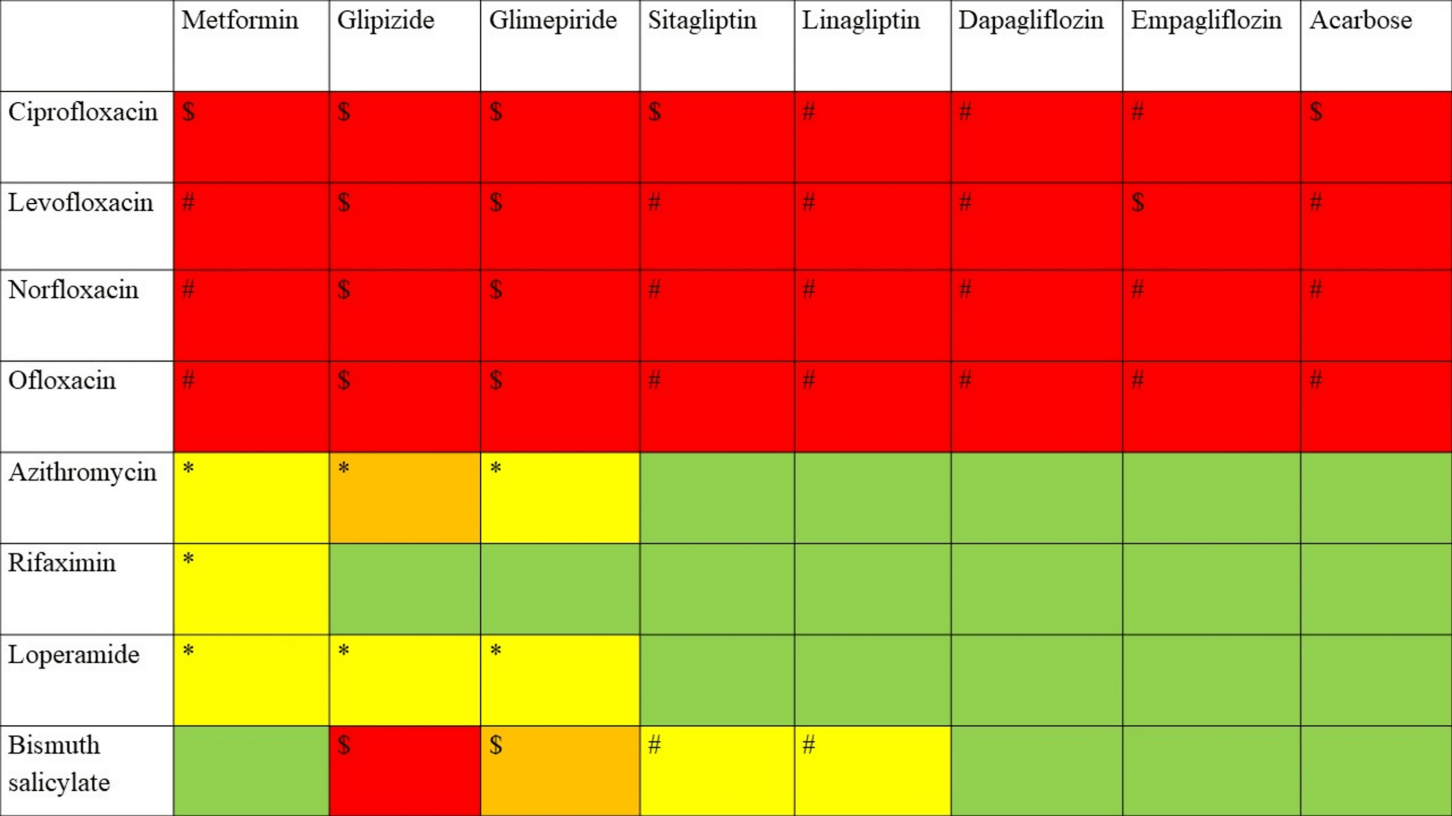
Traveler’s diarrhea drugs vs. antidiabetic drugs. The symbols and colors in the table denote different indications. *: mild interaction effects that imply no action is required. #: moderate interaction effects that mandate careful monitoring. $: severe interaction effects that suggest avoiding or using an alternate; green color: no interaction was identified; yellow color: interactions were discovered in a single database; orange color: interactions were identified in two databases; red color: interactions were detected in three to five databases

**Figure 2:**
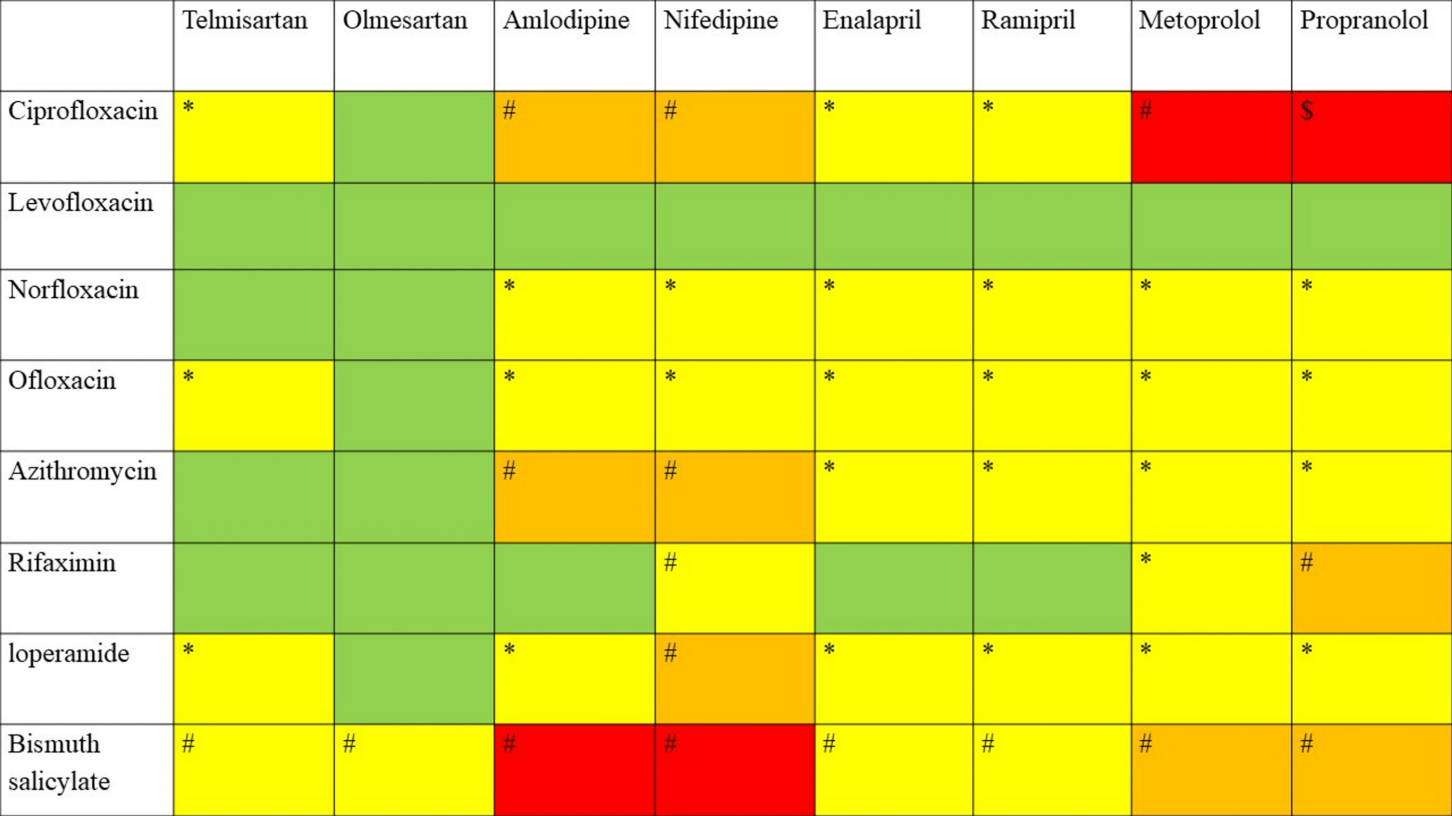
Traveler’s diarrhea drugs vs. antihypertensive drugs. The symbols and colors in the table denote different indications. *: mild interaction effects that imply no action is required. #: moderate interaction effects that mandate careful monitoring. $: severe interaction effects that suggest avoiding or using an alternate; green color: no interaction was identified; yellow color: interactions were discovered in a single database; orange color: interactions were identified in two databases; red color: interactions were detected in three to five databases

**Figure 3:**
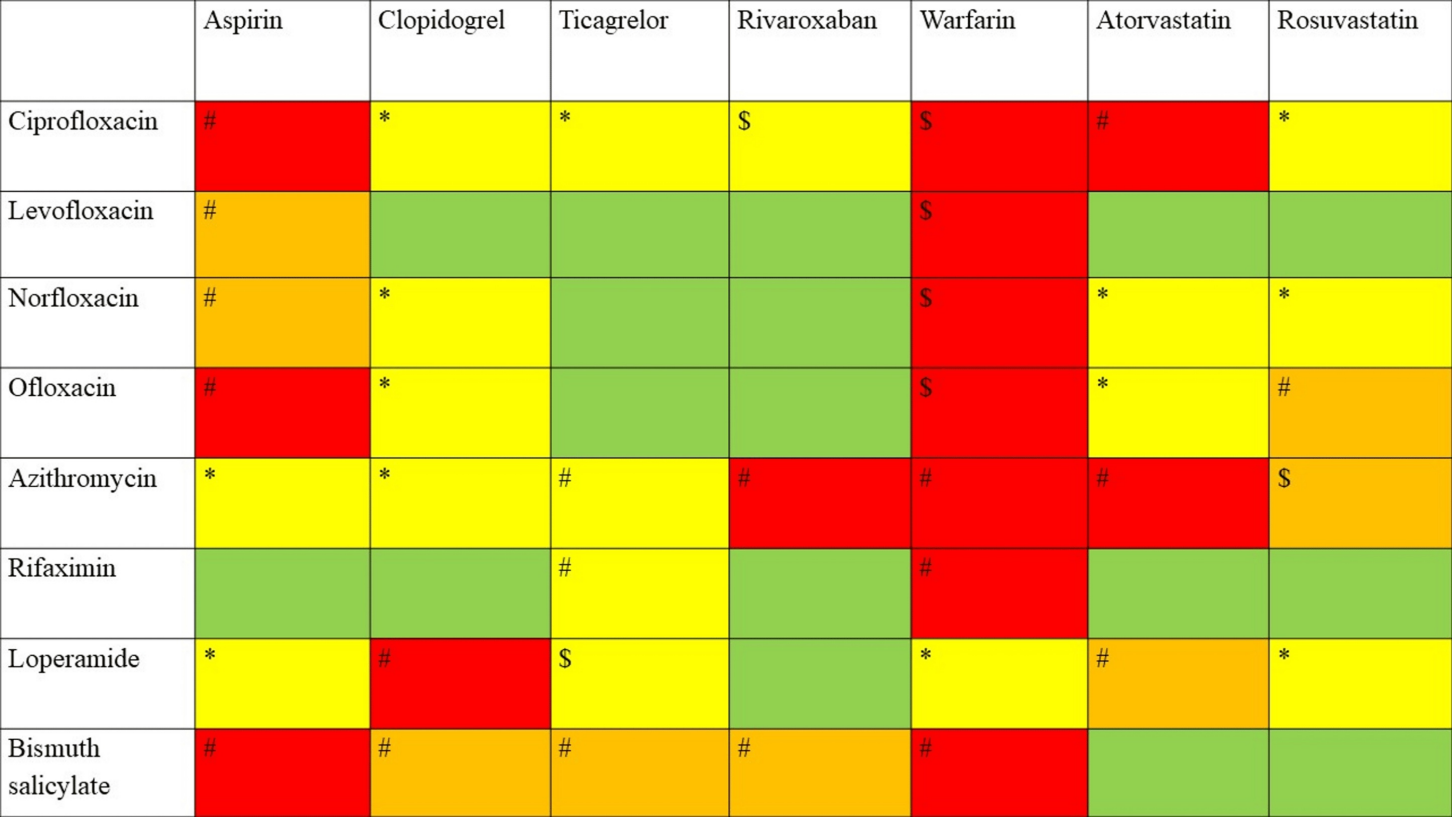
Traveler’s diarrhea drugs vs. cardioprotective drugs. The symbols and colors in the table denote different indications. *: mild interaction effects that imply no action is required. #: moderate interaction effects that mandate careful monitoring. $: severe interaction effects that suggest avoiding or using an alternate; green color: no interaction was identified; yellow color: interactions were discovered in a single database; orange color: interactions were identified in two databases; red color: interactions were detected in three to five databases

The most interaction potential was demonstrated by fluoroquinolones, notably ciprofloxacin, with sulfonylureas and warfarin, which could result in serious interactions that needed to be carefully managed. There were fewer notable interactions between azithromycin and rifaximin, which made them safer substitutes for patients with long-term coexisting illnesses. Due to its low interaction effects, levofloxacin was identified as a comparatively safer alternative for cardioprotective and hypertensive medications. The severity of the interactions was represented using symbols as mild, moderate, and severe. Mild interactions were determined to be unlikely to necessitate clinical intervention. For example, interactions that influenced drug metabolism only minimally or induced minor side effects without affecting therapeutic efficacy. Requiring monitoring or dosage adjustments were identified as moderate interactions. For instance, the interaction between metformin and ciprofloxacin may moderately impact blood glucose control, requiring consistent monitoring. Severe interaction included requiring alternate management or avoiding/using with caution. Significant clinical intervention, such as the substitution of medications or the complete avoidance of the combination, was indicated for severe interactions. For instance, the interaction between warfarin and ciprofloxacin is severe and elevates the risk of hemorrhage by inhibiting warfarin metabolism. This necessitates the close monitoring of international normalized ratio levels and potential dosage variations. The results of this review are presented in Figures 1-3.

Discussion

Interactions Between Drugs Used to Treat Traveler’s Diarrhea and Antidiabetic Drugs

Diabetes mellitus is a common comorbidity, and the use of antidiabetic drugs is widespread among affected individuals. Metformin is the only drug that showed interactions with all the drugs used in treating traveler’s diarrhea, irrespective of its severity. Fluoroquinolones disrupt glucose homeostasis by improving the release of insulin by interacting with antidiabetic agents, achieved by the inhibition of cytochrome P450 enzymes [9]. Almost all the databases revealed interactions between fluoroquinolones and antidiabetic drugs, with the severity ranging from moderate to severe. This reveals that fluoroquinolones should be prescribed with caution in individuals taking antidiabetic drugs. Quinolones can significantly alter blood glucose homeostasis by influencing the adenosine triphosphate-sensitive potassium channels found in pancreatic beta cells. Either hyperglycemia or hypoglycemia can be noted. Management could make dosage adjustments or use alternate options. The fluoroquinolones and sulfonylureas were found to produce severe interaction effects. Coadministration of both drugs elevates the restriction of pancreatic beta cells, thereby raising the risk of sulphonyl urea-induced hypoglycemia [10]. Bismuth salicylate interacted with sulfonylureas and dipeptidyl peptidase-4 inhibitors while the effect was severe and moderate, respectively. The moderate effect requires careful monitoring of blood glucose. We found azithromycin and loperamide to be safe, as they only exhibited mild interactions in one or two databases. Comparatively, rifaximin can be safer as it exhibits mild interaction only with metformin, whereas the other antidiabetic drugs showed no interaction effect.

Interactions Between Drugs Used to Treat Traveler’s Diarrhea and Antihypertensive Drugs

Hypertension is the most common chronic condition that necessitates long-term use of antihypertensive medications. Approximately 1.13 billion people are expected to be diagnosed with hypertension. The use of polypharmacy is more common among hypertensive individuals, as polypharmacy is associated with other comorbidities. Polypharmacy in hypertensive individuals is associated with an increased risk of drug interactions [11]. Distinct categories of antihypertensive medications are widely used. Angiotensin-converting enzyme inhibitors, angiotensin receptor antagonists, calcium channel blockers, and beta-blockers were chosen for this review [12]. Most of the interactions were found to be mild to moderate. Only the interaction between ciprofloxacin and propranolol was found to be major, and the effect has been shown in almost all the databases. Cytochrome 1A2 inhibitors, such as ciprofloxacin, can increase the level of propranolol. Only bismuth salicylate showed a moderate interaction with all antihypertensive medications, and most databases observed this effect. The combination of salicylates and antihypertensive medications can decrease their efficacy by inhibiting renal prostaglandins, sodium, and fluid retention. It can be managed by monitoring blood pressure. Nifedipine exerts mild-to-moderate interaction in most of the antidiarrheal medications. Ciprofloxacin, ofloxacin, and loperamide are the second category of drugs that exhibited mild-to-moderate interactions in most of the traveler’s diarrhea medications. Azithromycin, norfloxacin, and rifaximin exhibited mild interactions with some of these antihypertensive medications. No action is required for these mild interaction effects. Comparatively, levofloxacin was found to be the safest drug among hypertensive individuals, as it exerts no interaction effects with any of these antihypertensive medications.

Interactions Between Drugs Used to Treat Traveler’s Diarrhea and Cardioprotective Drugs

Cardiovascular diseases can be considered one of the leading causes of global burden. These days, the majority of travelers carry cardioprotective drugs as life-saving medications. Antiplatelet, anticoagulant, and antihyperlipidemic agents are the most common cardioprotective drugs in long-term use. Warfarin is the most cautious drug, as it exhibited moderate to severe interaction among most of the traveler’s diarrhea medications. The interactions were shown in all the databases. Fluoroquinolones are reported to produce interactions with warfarin, thereby elevating its anticoagulant effects. It is mandatory for an individual receiving both ciprofloxacin and warfarin to monitor prothrombin time. Careful monitoring and dosage adjustment are essential as the interaction between these medications can increase the prothrombin time [13]. The interaction effect was found to increase warfarin levels for displacing warfarin from protein-binding sites, resulting in the alteration of vitamin K and clotting factor-producing gut flora. Fluoroquinolones are known to restrict the metabolism of cytochrome P450 and reduce the clearance of medications possessing a narrow therapeutic index, such as warfarin [14]. Ciprofloxacin is the only drug that exerts interaction with all cardioprotective drugs. However, it exhibits major interaction with warfarin and rivaroxaban, whereas it exhibits moderate interaction with aspirin and atorvastatin. The major interaction effect of ciprofloxacin is that it can inhibit the hepatic metabolism of the other antihypertensive drug, thereby increasing the risk of bleeding. The management of these interactions entails either monitoring International normalized ratio levels or selecting an alternate drug. Additionally, azithromycin showed moderate-to-severe interactions with all these cardioprotective drugs. Moreover, certain drugs such as norfloxacin, ofloxacin, loperamide, and bismuth salicylate exhibit moderate interactions with the majority of cardioprotective drugs. Comparatively, levofloxacin and rifaximin are considered to be safe, as these drugs interact with only two cardioprotective drugs. As the interaction effects are moderate, monitoring the INR levels is required to avoid the bleeding risk.

Limitations

One limitation of this review is the focus on interactions between traveler’s diarrhea medications and drugs for chronic conditions such as diabetes, hypertension, and cardiovascular disease. This review did not include proton pump inhibitors (PPIs), which are often co-prescribed for gastrointestinal protection. Antidepressants and antiepileptics, which have considerable interaction potential, were not covered in the review. Finally, the study did not include clinical evidence or a systematic review.

## Conclusions

This review focuses on the interaction effect of medications used in traveler’s diarrhea with chronic conditions such as antidiabetic, antihypertensive, and cardioprotective medications. This review features the importance of a precise approach in prescribing medications for traveler’s diarrhea, especially for patients with chronic comorbidities. Considering ciprofloxacin in fluoroquinolones requires caution as it exerts significant interactions in antidiabetic and cardioprotective treatments. Levofloxacin and rifaximin were found to be the better options, as they exhibit only minimal interactions. These discussions play a pivotal role in improving awareness and providing tailored treatment for the interaction, ensuring patient well-being.
